# Tumor necrosis factor polymorphisms associated with tumor necrosis factor production influence the risk of idiopathic intermediate uveitis

**Published:** 2013-01-28

**Authors:** Denize Atan, Jarka Heissigerova, Lucia Kuffová, Aideen Hogan, Dara J. Kilmartin, John V. Forrester, Jeff L. Bidwell, Andrew D. Dick, Amanda J. Churchill

**Affiliations:** 1 School of Clinical Sciences, Bristol Eye Hospital, Lower Maudlin Street, Bristol, BS1 2LX, United Kingdom; 2 Institute of Medical Sciences, University of Aberdeen, Foresterhill, Aberdeen, AB25 2ZD, United Kingdom; 3 Research Foundation, Royal Victoria Eye and Ear Hospital, Adelaide Road, Dublin 2, Republic of Ireland; 4 School of Cellular & Molecular Medicine, University of Bristol, School of Medical Sciences, Bristol, BS8 1TD, United Kingdom

## Abstract

**Purpose:**

Idiopathic intermediate uveitis (IIU) is a potentially sight-threatening inflammatory disorder with well-defined anatomic diagnostic criteria. It is often associated with multiple sclerosis, and both conditions are linked to HLA-DRB1*15. Previously, we have shown that non-infectious uveitis (NIU) is associated with interleukin 10 (*IL10*) polymorphisms, *IL10-2849A* (rs6703630), *IL10+434T* (rs2222202), and *IL10+504G* (rs3024490), while a *LTA+252AA/TNFA-238GG* haplotype (rs909253/rs361525) is protective. In this study, we determined whether patients with IIU have a similar genetic profile as patients with NIU or multiple sclerosis.

**Methods:**

Twelve polymorphisms were genotyped, spanning the tumor necrosis factor *(TNF)* and *IL10* genomic regions, in 44 patients with IIU and 92 population controls from the UK and the Republic of Ireland.

**Results:**

IIU was strongly associated with the *TNFA-308A* and *TNFA-238A* polymorphisms. We found the combination of *TNFA-308* and *-238* loci was more strongly associated with IIU than any other loci across the major histocompatibility complex, including *HLA-DRB1*.

**Conclusions:**

*TNF* polymorphisms, associated with increased TNF production, are highly associated with IIU. These results offer the potential to ascribe therapeutic response and risk (i.e., the influence of HLA-DRB1*15 status and *TNFR1* polymorphism) to anti-TNF therapy in IIU.

## Introduction

Intermediate uveitis is an anatomically defined diagnosis, reserved for patients who have intraocular inflammation primarily involving the vitreous, peripheral retina, and pars plana [1]. Intermediate uveitis is a common presentation to general ophthalmology practice, particularly among children [2], with a population prevalence of 1.4/100,000 [3]. Generally, the onset is insidious with symptoms of blurred vision and floaters, and remission is infrequent and transitory. Vision loss is most commonly caused by chronic cystoid macular edema or secondary glaucoma [4].

The diagnosis of idiopathic intermediate uveitis (IIU) is restricted to patients in whom there is no evidence of infection; pars planitis refers to a subset with concomitant snowball formation or pars plana exudation (snowbanking) [2]. The presenting clinical phenotype overlaps with infectious causes of uveitis, including syphilis, tuberculosis, Lyme disease, cat-scratch fever, toxoplasmosis, Whipple's disease, Epstein-Barr virus, human T lymphotropic virus type I, and human immunodeficiency virus. Furthermore, intermediate uveitis is commonly associated with several diseases that span the full spectrum of the autoimmune-autoinflammatory (AI) immunological disease continuum [5], such as sarcoidosis, multiple sclerosis (MS), thyroid disease, and inflammatory bowel disease (IBD) [6]. Moreover, the familial aggregation of cases with IIU and other AI diseases suggests the existence of common genetic variants that underlie susceptibility to AI disease and/or a common environmental agent [7-13]. While unconfirmed, a general hypothesis is that an infectious foreign agent (virus or bacterium) systemically activates self-reactive T-cells in genetically susceptible individuals. The mechanisms by which this occurs are likely to involve molecular mimicry or non-specific bystander activation of self-reactive T-cells that home to the eye, leading to chronic, relapsing, or recurrent intraocular inflammatory disease [14].

The connection between IIU and MS merits further interrogation because both have been associated with the HLA-DRB1*15 antigen, a subtype of DR2 [15,16]. MS develops in 14% to 16.2% of patients with IIU, and either disease can precede the other [16,17]. In one study, 31% of patients with IIU who were HLA-DRB1*15^+^ also had MS, and 25% had a positive family history of MS [15]. Patients with MS have autoreactive T-cells and antibodies directed against glial proteins, such as myelin basic protein, that are associated with actively demyelinating lesions [18]. Glial proteins are also detected in snowbanks [8]. The inference is that patients with MS and IIU have autoreactive T-cells directed toward a common glial epitope.

The experimental models of uveitis (experimental autoimmune uveoretinitis, EAU) and MS (experimental autoimmune encephalomyelitis, EAE) share many common features; in particular, the detrimental role of tumor necrosis factor-α (TNFα). A consistent feature of EAU is the increased TNFα expression found in inflammatory cell infiltrates [19,20], and similarly, high levels of TNFα are found in MS lesion sites in patients with MS [21]. Furthermore, cerebrospinal fluid levels of TNFα are increased in chronic progressive MS compared with controls and correlate with disease severity [22]. Consequently, anti-TNFα monoclonal antibodies and a TNF receptor 1:immunoglobulin G fusion protein to ameliorate disease were tested in EAU and EAE with promising results [23,24]. Although anti-TNFα therapies translate effectively to the clinic for uveitis [25], this is not the case for patients with MS in whom anti-TNFα agents worsen disease and precipitate demyelination in others [26]. As a result, the administration of anti-TNFα therapy to patients with IIU has been somewhat more tentative. However, treatment successes with anti-TNFα therapy for patients with IIU have been reported [27,28], while others have described episodes of central nervous system demyelination in patients with IIU for the first time following anti-TNF therapy [29-31].

Previously, we have shown that specific genotypes in three haplotype-tagging single nucleotide polymorphisms (htSNPs) in the *IL10* gene (rs6703630, rs2222202, and rs3024490) are significantly associated with susceptibility to non-infectious uveitis (NIU), while a *LTA+252AA/TNFA-238GG* haplotype (rs909253 and rs361525) is protective [7]. Moreover, patients with two closely overlapping white dot syndromes, punctuate inner choroidopathy (PIC) and multifocal choroiditis with panuveitis (MFCPU), demonstrated identical associations with the *IL10* haplotype, *IL10-2849AX/+434TC,* that were not observed in other subgroups [32]. MFCPU and PIC fall under the SUN (Standardized Uveitis Nomenclature) Working Group classification of posterior uveitis with clinical evidence of multifocal choroiditis [1], and both disorders are characterized by inflammatory microgranulomata at the chorioretinal interface [33]. Hence, our data on the genetic profile of these patients suggested that MFCPU and PIC may be manifestations of the same disease [32].

In this report, we sought to further interrogate our study population to determine whether patients with IIU (which is also defined anatomically by the SUN Working Group) demonstrate a characteristic genetic profile that differs from that of patients with non-specific NIU. In addition, we were interested to know whether this profile was similar to genetic associations identified previously in patients with MS.

## Methods

### Subjects

One hundred and thirty-six subjects in good general health (45 male, 91 female; age range 21-89 years’ old) were recruited from three regional centers in Bristol (Bristol Eye Hospital), Aberdeen (Grampian University Hospitals), and Dublin (The Royal Victoria Eye and Ear Hospital) as part of a larger study [7]. Ethical approval was given by each center, and the study adhered to the tenets of the Declaration of Helsinki. All subjects were white Caucasians of British or Irish descent for at least two generations.

Informed consent was obtained from all participants (44 patients, 92 controls), after the nature and possible consequences of the study were explained. All subjects were given a full ophthalmic examination for diagnostic evaluation according to the guidelines of the SUN Working Group [1]. All patients managed at the three regional centers had routine diagnostic and pretreatment investigations as part of the previous study [7]. Forty-four patients diagnosed with IIU were included in the study, of whom nine had pars planitis characterized by snowbanking. They were consecutively recruited over a five-year period between 2002 and 2007 from regional uveitis clinics at the three centers. Patients with coexisting MS were excluded, as were patients with any underlying etiology for intermediate uveitis (e.g., sarcoidosis) based on their pretreatment investigations. Control subjects were examined to ensure that they had no evidence of preexisting inflammatory eye disease. They were excluded if they had any eye-specific disorder or systemic disease with a significant immunogenetic etiology, including known associations with cytokine gene polymorphisms (e.g., age-related macular degeneration, glaucoma, type 1 diabetes mellitus [T1D], ankylosing spondylitis, rheumatoid arthritis, systemic lupus erythematosus, chronic obstructive pulmonary disease, ischemic heart disease, neoplasia).

### Genotype associations were determined for three parameters of disease severity

1. Ocular remission, using SUN guidelines [1].

2. Maintenance immunosuppression, defined as the most recent combination of immunosuppressants to consistently control disease activity for at least three months, with no increase in immunosuppression during this period (more fully described elsewhere) [7].

3. Visual outcome, assessed by (a) visual acuity (VA) at the census date and (b) change in VA from disease onset to the census date (defined, according to SUN guidelines, as a decrease in Snellen VA of >3 lines) [1].

### Genotyping

HtSNPs in the *IL10* and *TNF* genomic regions were selected and genotyped using published methods [7], and Hardy–Weinberg probabilities were calculated for the larger cohort [7]. The *TNFd* microsatellite polymorphism was genotyped as previously described [34,35]. HLA class I (A, B, and C) and II (DRB1 and DQB1) typing was performed using sequence specific primers (SSP–PCR) at medium resolution [36]. Sequence accession numbers were NT_021877 for *IL10* and NT_007592 for *TNFA*.

### Statistical analyses

Demographic information, clinical course parameters, patient, and control genotype distributions were compared between dichotomous groups using the two-tailed χ^2^ test (chi-square) or Fisher’s exact test where appropriate, using SPSS 14.0 (SPSS UK Ltd, Woking, UK) and UNPHASED [37]. Snellen VAs were converted to logMAR for analyses. Distributions of ordinal phenotypic characteristics were compared using the Kruskal–Wallis non-parametric test, and for continuous characteristics using the two-tailed Student *t* test in SPSS.

Associations across the major histocompatibility complex (MHC) were determined using UNPHASED [37]. UNPHASED uses an expectation-maximization (EM) algorithm to perform the likelihood ratio X^2^ test on case-control data with the advantage that this algorithm can handle multiallelic MHC data [37]. The genetic models that best explained significant genetic associations with IIU were determined using PLINK 1.07 [38]. Haplotype associations were also determined in PLINK, which is limited to biallelic SNPs.

The Bonferroni correction was applied to genotypic data to adjust for the number of comparisons (n=total number of loci or haplotypes) and assumes that the statistical comparisons are independent. Odds ratios were calculated in OpenEpi version 2.3.1 [39]. Since the population prevalence of IIU is relatively low at 1.4/100,000 in a European cohort similar to the UK [3], we predicted the relative risk of IIU to approximate the odds ratio.

Sample sizes were calculated using OpenEpi based on the published minor allele frequencies in a European cohort of the SNPs (HapMap CEU cohort) and *TNFd* microsatellite polymorphisms (a UK cohort) under investigation [35,40]. Based on these published data, our study had 80% power to detect differences in allele frequency between patients and controls with a minimum odds ratio of 3.0 and with 95% confidence levels.

## Results

The demographics of the IIU and control groups were similar for age and sex with no significant differences (Table 1). As we have found previously, there was a high prevalence of AI disease in patients’ self-reported personal medical histories (29.5% versus 0% in controls) and family history (20.5% versus 5.4% in controls) [7]. The conditions that patients reported in their personal histories were non-specified arthritis/arthralgia (four patients), asthma (three patients), primary hyperthyroidism/hypothyroidism (two patients), primary hypoparathyroidism (one patient), fibromyalgia (one patient), psoriasis and arthritis (one patient), and type 2 diabetes mellitus (T2D, one patient). In the family histories, only one patient had a relative with MS, and another had a family member who was also affected by IIU. Other conditions reported by patients in their family history were T2D (one patient), celiac disease (one patient), rheumatoid arthritis (two patients), non-specified arthritis (one patient), inflammatory bowel disease (one patient), and primary hyperthyroidism (one patient). Five controls had a family history of AI disease: four were unaffected relatives of patients in the study, and one had a strong family history of arthritis.

**Table 1:** Demographic information on recruited subjects

Uveitis classification	Number in group	Age at recruitment (years)		Sex	
		Mean +/− SD	Range	Male	Female
IIU patients	44	43.2 +/− 14.6	22–87	18	26
Healthy controls	92	48.9 +/− 16.9	21–89	27	65
Comparison of IIU versus control groups		p=0.055		p=0.182	

The IIU and control cohorts were statistically similar in mean age, age range and sex distribution. The two groups were compared using the 2-tailed unpaired *t* test for age and 2-tailed X^2^ test for sex. Abbreviations: IIU, idiopathic intermediate uveitis; SD, standard deviation.

### Idiopathic intermediate uveitis is associated with *tumor necrosis factor* polymorphisms

*Lymphotoxin alpha* (*LTA*), *TNFA*, and *TNFd* are located within the class III region of the MHC on chromosome 6p21.3. Hence, we used UNPHASED to analyze associations between loci across this region and IIU since UNPHASED can handle data from multiallelic SNPs and can model associations to determine which loci are primarily associated with disease among several linked loci. We also included SNPs within the *IL10* complex on chromosome 1.

Using UNPHASED, we found that loci *TNFA-308*, *TNFA-238*, *HLA-DRB1*, and *HLADQB* were associated with IIU, but only the associations with *TNFA-308* and *TNFA-238* remained significant after correction for multiple comparisons (Table 2). Since a specific *TNFA* promoter allele contains *TNFA-308A*, and a *LTA* promoter allele contains *LTA+252G* in several combined *HLA-TNFA-LTA* haplotypes, including the HLA 8.1 ancestral haplotype (A1, B8, DR3) [41], and since HLA-DRB1*17 is a subtype of HLA-DR3, we looked for a similar combined *HLA-TNFA-LTA* haplotype demonstrating an association with IIU. Nonetheless, no combined haplotype demonstrated significant association with disease in our cohort (data not shown). Moreover, in further modeling analyses in UNPHASED, we conditioned on *TNFA-308*, *TNFA-238*, *HLA-DRB1*, and *HLA-DQB*, either singly or combination. In these analyses, the combination of loci, *TNFA-308* and *TNFA-238*, was the most significantly associated with IIU throughout (p<0.00001), suggesting that the associations were explained solely by these two loci. Although HLA-DRB1*15 (17.9% versus 15.5% in controls, p_uncorr_=0.63) and HLA-DRB1*17 (19.1% versus 10.9% in controls, p_uncorr_=0.07) were the most prevalent *HLA-DRB1* alleles, individually they were not significantly associated with disease in this cohort.

**Table 2:** Associations between IIU and *HLA*, *TNF* and *IL10* loci.

Locus	Chromosome	X^2^	Degrees of freedom	p value	p_c_ value
*IL10 −3545*	1	0.129	2	0.7195	NS
*IL10 −2849*	1	1.186	2	0.2762	NS
*IL10 −1082*	1	0.343	2	0.5579	NS
*IL10 −819*	1	0.426	2	0.5138	NS
*IL10 +434*	1	0.021	2	0.8850	NS
*IL10 +504*	1	0.004	2	0.9472	NS
*IL10 +1847*	1	0.610	2	0.4346	NS
*LTA+252*	6	2.992	2	0.0837	NS
*TNFA −308*	6	10.99	2	0.0009	0.0171
*TNFA −238*	6	11.54	2	0.0007	0.0133
*TNFA +488*	6	0.494	2	0.4821	NS
*TNFd*	6	1.688	10	0.8904	NS
*HLA A*	6	18.94	15	0.2165	NS
*HLA Cw*	6	13.15	12	0.3581	NS
*HLA B*	6	27.06	22	0.2088	NS
*HLA Bw*	6	0.001	1	0.9701	NS
*HLA DRB1*	6	28.45	12	0.0048	NS
*HLA DR3–5*	6	1.194	3	0.7953	NS
*HLA DQB*	6	15.22	6	0.0254	NS

IIU is significantly associated with the *TNFA-308* and *TNFA-238* loci. There were associations with the *HLA DRB1* and *HLA DQB* loci that lost significance after Bonferroni correction. Statistical analyses used the Likelihood ratio χ^2^ test in UNPHASED with 2-tailed probability values and Bonferroni correction for the number of loci (n=19). Abbreviations: NS, not significant with a p value>0.05; p value, uncorrected probability value; p_c_, Bonferroni corrected p value (p_c_=p x19); X^2^, Chi-square test.

The associations between IIU and the *TNFA-308* (rs1800629) and *TNFA-238* (rs361525) loci were best explained by an allelic (additive) genetic model in which the minor alleles, *TNFA-308A* (p_c_=0.0042) and *TNFA-238A* (p_c_=0.0019), were significantly associated with IIU (Table 3). Analyses using either a dominant or recessive model for the associations between *TNFA-308* and *TNFA-238* with IIU in PLINK were not significant (data not shown).

**Table 3:** Associations between IIU and the *TNFA-308* and *TNFA-238* loci are most compatible with an additive (allelic model)

Locus	IIU patients	Healthy controls	X^2^	Degrees of freedom	p value	p_c_ value	Odds ratio for minor allele (95% CI)
*TNFA −308* (rs1800629)							
Genotype frequency AA/AG/GG	3/24/17	2/24/66	13.84	2	0.001	0.019	-
Allele frequency A/G	30/58	28/156	12.64	1	0.00038	0.0042	2.9 (1.6–5.2)

*TNFA −238* (rs361525)							
Genotype frequency AA/AG/GG	0/16/28	1/6/85	18.36	2	<0.0010	<0.019	-
Allele frequency A/G	16/72	8/176	14.16	1	0.00017	0.0019	4.9 (2.0–11.9)

Modeling the genetic association revealed an underlying allelic (additive) model with the minor alleles, *TNFA-308A* and *TNFA-238A*, associated with disease. Dominant and recessive models were not significant. Statistical analyses of genetic model used the Pearson X^2^ test with 2-tailed probability values in PLINK with Bonferroni correction for the number of loci (n=19). Genotype associations were determined using the Fisher exact test in SPSS with Bonferroni correction (n=19). Odds ratios were calculated in Open Epi. Abbreviations: p value, uncorrected probability value; p_c_, Bonferroni corrected p value (p_c_=p x11); X^2^, Chi-square test; CI, confidence intervals.

Given the allelic associations between the *TNFA-308* and *TNFA-238* loci with IIU, and the significant association between the two combined loci and IIU in our modeling analyses in UNPHASED, we investigated combined allelic haplotypes of the two loci. In these analyses, we found the *TNFA-308G/TNFA-238G* haplotype was the most significantly protective haplotype (50.9% in patients versus 80.4% in controls, p_c_=000004), i.e., negatively associated with disease (Table 4). No *IL10* SNP genotypes were associated with disease after correction for multiple comparisons (Table 2).

**Table 4:** Association between combined *TNFA-308* and *TNFA −238* haplotypes with IIU

*TNFA-308/-238* haplotype	Inferred frequency in IIU patients	Inferred frequency in controls	X^2^	Degrees of freedom	p value	p_c_ value
GG	0.509	0.804	24.31	1	0.000001	0.000004
GA	0.145	0.042	8.896	1	0.002858	0.011432
AG	0.312	0.152	9.132	1	0.002512	0.010048
AA	0.033	0.002	4.794	1	0.028560	NS

The combined *TNFA-308G/-238G* haplotype had a significant *negative* association with IIU, suggesting a protective effect against development of the disease. Two marker haplotypes were inferred in PLINK. Statistical analyses used the Pearson X^2^ test with 2-tailed probability values in PLINK with Bonferroni correction for the number of haplotypes (n=4). Abbreviations: %, percentage; IIU, idiopathic intermediate uveitis; p value, uncorrected probability value; p_c_ value, Bonferroni corrected p value; NS, not significant with a p value>0.05.

### *Tumor necrosis factor* and *interleukin 10* polymorphisms are not associated with severity of disease

There were no significant genotype associations with our three parameters of disease severity after correction for multiple comparisons, including (i) ocular remission, (ii) the requirement for and level of maintenance immunosuppression, and (iii) visual outcome in terms of absolute logMAR VA and decrease in VA of >3 lines at the census date. Since other investigators have found a correlation between the *IL10-1082AA* genotype and poor visual outcome in patients with IIU (VA<6/12 in both eyes while quiescent, five years after presentation) [42], we asked whether there was a similar correlation in our cohort of patients. In fact, there was no association between the *IL10-1082* genotype and visual outcome (p_uncorr_=0.113 for VA decrease >3 lines with Fisher’s exact test; p_uncorr_=0.796 for logMAR VA at census date with the Kruskal–Wallis test). Of 38/44 IIU patients with bilateral disease, seven patients experienced a decrease in visual acuity by >3 lines in at least one eye and one patient in both eyes, between disease onset and the census date, an average follow-up period of 10.6 years (range 4.7 to 36.5 years). The latter was the only patient to have a Snellen VA <6/12 in both eyes at the census date due to glaucoma and chronic cystoid macular edema, despite previous treatment with high-dose prednisolone, cyclosporine, tacrolimus, mycophenolate mofetil, azathioprine, and methotrexate. This patient had an *IL10-1082AG* genotype. In fact, no patients with a VA<6/12 in at least one eye or decrease in VA>3 lines by the census date had an *IL10-1082AA* genotype.

## Discussion

The results of this study have shown that IIU is strongly associated with polymorphisms of the *TNFA-308* and *-238* loci and that these associations appear to be independent of HLA-DRB1*15 and the ancestral haplotype, HLA-A1, B8, DR3. The relationship is best explained with an allelic model, in which the *TNFA-308A* and *TNFA-238A* minor alleles are associated with IIU. Moreover, the combined *TNFA-308G/-238G* haplotype confers resistance to IIU (p_c_=0.000004). Collectively, these results are enticing since the *TNFA-308* and *TNFA-238* loci have been shown to influence TNF production levels and have been associated with several other diseases on the AI immunological disease continuum [43].

Our results are consistent with previous work that has shown that the *LTA+252AA/TNFA-238GG* haplotype is negatively associated with non-infectious uveitis (p=0.00031) in a cohort where patients with six different uveitic syndromes contributed equally to the analyses [7]. Moreover, two white dot syndromes, PIC and MFCPU, demonstrated an extended *LT-TNF* haplotypic association with disease [32]. Yet none of the other uveitic syndromes in our original study, including Behçet’s disease, sarcoidosis, and sympathetic ophthalmia, demonstrated significant associations with the *TNFA-238* and *TNFA-308* loci that were independent of their HLA associations, for example, sympathetic ophthalmia with HLA-DRB1*04 [7,32,44](data not shown). *HLA-B27* is a well-recognized susceptibility allele for idiopathic anterior uveitis and linkage disequilibrium between *HLA-B27* and other variants within the human MHC known to confound analyses for additional susceptibility loci in this region. Several previous studies investigating the prevalence of *TNF* polymorphisms in patients with anterior uveitis have suggested genetic associations, but the inconsistent outcomes may be a result of heterogenous patient cohorts with a mixed number of underlying systemic disease associations or incomplete stratification analyses for *HLA-B27* in patient and control groups [45-47].

The investigation of genetic influences on TNF production is also complicated by the location of the *TNF* gene cluster within the MHC, the most polymorphic region of the human genome [48]. Only 1.2 kb separates the polyadenylation site of *LTA* and the transcription start site of *TNFA* within the *TNF* gene cluster of the MHC class III region, genes that encode the structurally homologous inflammatory cytokines, LTα and TNFα. Moreover, examples of long-range linkage disequilibrium (LD), extending more than 2 Mb, arise in a subset of MHC haplotypes because of LD between tightly linked segments of strong LD creating a unique microstructure [49,50]. Consequently, the *TNFA-308A* polymorphism is often linked to the *LTA+252G* polymorphism in several combined *HLA-TNF-LT* haplotypes, including the HLA 8.1 ancestral haplotype (A1, B8, DR3) [41] that has repeatedly been associated with higher TNF production levels and many immunopathological diseases [43,51-57].

Why some studies have failed to demonstrate a correlation between polymorphisms of *TNFA* and TNFα production levels might be partly attributed to the chance representation of different subsets of haplotypes in each study, and partly due to differences in experimental methods, including the cell-type investigated, culture conditions, type of stimulant used (if any), and means of measurement [56]. Analyzing chromatin structure is one method for determining regions of a gene involved in transcriptional activation, and DNase I hypersensitivity (HS) sites represent nucleosome-free regions of a gene that are accessible to transcription factors as well as the DNase I endonuclease [58-60]. DNase I HS sites within regions of a gene demonstrating a high degree of DNA sequence conservation with other species, known as conserved non-coding sequences, are most likely to represent important regulatory sequences. Consequently, it is relevant that the proximal *TNFA* and *LTA* promoters are highly conserved between species, and constitutively active DNase I HS sites have been identified in these regions in human monocyte and T-cell lines, whereas other inducible HS sites are cell-type and stimulus dependent [61-66]. Although further regulatory elements have been identified in conserved non-coding sequences elsewhere in the *TNF* gene cluster [62,67-71], the *LTA* and *TNF* proximal promoter regions attain the highest conservation scores. Moreover, the *TNFA-308* polymorphism appears to affect transcription factor binding and *TNF* transcriptional activity in a cell-type and stimulus-specific fashion [72]. These data from functional chromatin studies combined with the evidence from disease association studies further implicate this region of the MHC in the immunopathogenesis of several AI diseases. However, the relevance of specific polymorphisms in disease pathogenesis likely depends on the importance of the cell-type and stimulatory conditions in which the polymorphisms have most influence. Although one limitation of this study is that we did not include an analysis of TNF production levels based on patient genotype, this would clearly require a systematic investigation of the response of different leucocyte subclasses to varying stimuli before and after systemic immunosuppression is administered, which is beyond the scope of this report.

Perhaps for this reason, the role of TNF in the pathogenesis of AI disease is not always straightforward. The timing and duration of TNF expression are important in determining the pathogenic versus protective roles of TNF, since prolonged TNF exposure can activate antigen-presenting cells (APCs), augment antigen-presentation capability, and upregulate the expression of costimulatory molecules, while in other situations, TNF can inhibit the function of mature dendritic cells (DCs), induce their apoptosis, and impair antigen presentation [73]. In addition, exposure of DCs to TNFα in vitro has the capacity to induce a tolerogenic “semimature” functional phenotype on these cells, which can themselves secrete further TNFα to act in an autocrine fashion. When administered in vivo, TNFα-activated semimature DCs critically depend on the microenvironment to determine whether they remain tolerogenic or become immunogenic [74-77]. Hence, APCs are influenced by environmental cytokine signals that can promote either immunity or tolerance depending on their timing. These data might explain the paradoxical effects of anti-TNF therapy in MS. In comparison to IIU, the HLA class II region contributes most to genetic susceptibility to MS by linkage, case-control, and genome-wide association studies [78-82], while the link between *TNF* polymorphisms and MS remains contentious: *TNFA-308A* was significantly associated with reduced risk of MS in one large meta-analysis [83], but not another [84]. Since anti-TNF therapy for Crohn’s disease and rheumatoid arthritis (conditions not known to be linked to MS) have been reported to precipitate subsequent demyelination [85-88], low levels of TNF per se, whether due to TNF blockade or genetic factors, may be the major risk for demyelination [89], and systemic anti-TNF therapy for IIU might present the same risk. MS patients with a TNF receptor 1 (*TNFR1*) polymorphism, rs1800693, may be at particular risk of an adverse response to anti-TNF treatment because this SNP results in a soluble TNFR1 isoform that naturally antagonizes the action of TNF [89]. Whether this SNP is also predictive of demyelination after anti-TNF therapy in patients with IIU remains to be determined. Nevertheless, the data suggest that TNF has different roles in initiating and maintaining the two disorders: high levels increase the risk of IIU, and low levels increase the risk of demyelination; in addition, these levels are determined by several genetic factors, such as *TNF* and *TNFR1* polymorphisms, and environmental factors such as exposure to anti-TNF therapy.

In comparison, *IL10* polymorphisms appeared to have less influence on IIU susceptibility in our cohort. IL10 has mainly anti-inflammatory properties: it downregulates the expression of MHC class II and costimulatory molecules, inhibits the maturation of DCs, and inhibits the release of proinflammatory cytokines, resulting in the suppression of Th1 cell responses [90-93]. Furthermore, IL10 production by antigen-specific CD4^+^ Tregs is enhanced by IL10 [94]. Nevertheless, we did not detect an association between *IL10-1082AA* and poor visual outcome, as reported previously [42]. Neither was there an association between *IL10-1082* and change in visual acuity from disease onset to the census date. One caveat is that 33/102 patients in the previous report had a Snellen VA <6/12 in both eyes after five years of follow-up [42], while only 1/44 patients met these criteria in our cohort. Although we predicted a change in visual acuity would be a better predictor of disease severity, it is likely that both visual outcome measures are confounded by coexisting ocular disease, differences in treatment regimen, and response to treatment between patients.

A further limitation of this study is that patients with coexistent MS were excluded from the outset, and none of our cohort developed MS during the follow-up period. Furthermore, only 2/44 patients were on anti-TNF therapy during the period of the study, and neither patient developed central nervous system demyelination. Nevertheless, the strong association of *TNF* haplotypes with IIU demonstrates the future need to investigate further their correlation with TNF expression, disease severity, and response to treatment with anti-TNF agents. Additionally, the association is important in driving a future ability to tailor therapy to those who will benefit, and to investigate further the relationship in the context of HLA-DRB1*15 status and *TNFR1* polymorphism, to direct treatment to those that will demonstrate maximum benefit, but also to ensure no adverse effects (as demonstrated in MS).
